# Photoluminescence of InAs/GaAs quantum dots under direct two-photon excitation

**DOI:** 10.1038/s41598-020-67961-z

**Published:** 2020-07-02

**Authors:** Xian Hu, Yang Zhang, Dorel Guzun, Morgan E. Ware, Yuriy I. Mazur, Christoph Lienau, Gregory J. Salamo

**Affiliations:** 1 Institute for Nanoscience and Engineering, University of Arkansas, Fayetteville, AR 72701 USA; 2 Department of Electrical Engineering, University of Arkansas, Fayetteville, AR 72701 USA; 3 Institute of Physics and Center of Interface Science, Carl Von Ossietzky University, 26129 Oldenburg, Germany

**Keywords:** Quantum dots, Nonlinear optics

## Abstract

Self-assembled quantum dots grown by molecular beam epitaxy have been a hotbed for various fundamental research and device applications over the past decades. Among them, InAs/GaAs quantum dots have shown great potential for applications in quantum information, quantum computing, infrared photodetection, etc. Though intensively studied, some of the optical nonlinear properties of InAs/GaAs quantum dots, specifically the associated two-photon absorption of the wetting and barrier layers, have not been investigated yet. Here we report a study of the photoluminescence of these dots by using direct two-photon excitation. The quadratic power law dependence of the photoluminescence intensity, together with the ground-state resonant peak of quantum dots appearing in the photoluminescence excitation spectrum, unambiguously confirms the occurrence of the direct two-photon absorption in the dots. A three-level rate equation model is proposed to describe the photogenerated carrier dynamics in the quantum dot-wetting layer-GaAs system. Moreover, higher-order power law dependence of photoluminescence intensity is observed on both the GaAs substrate and the wetting layer by two-photon excitation, which is accounted for by a model involving the third-harmonic generation at the sample interface. Our results open a door for understanding the optical nonlinear effects associated with this fundamentally and technologically important platform.

## Introduction

With a high level of growth controllability and a superior optical activity, self-assembled InAs/GaAs quantum dots (QDs) grown by molecular beam epitaxy (MBE) have become a promising testbed intensively explored for many fundamentally and technologically important fields, such as quantum information^1,2^, quantum computing^3,4^, plasmonics^5^, infrared photodetection^6,7^, integrated photonics^8–10^, and solar energy harvesting^11,12^. For example, single photon sources based on InAs/GaAs QDs have demonstrated unrivalled performance in terms of single photon purity, indistinguishability, and brightness^13–17^, and thus hold great promise for quantum information and communication applications. On the other hand, atomic-like electronic states in InAs QDs naturally embody rich quantum physics that enables the manipulation of charge and spin degrees of freedom of either electrons or holes down to the single particle level^18,19^. Things become even more intriguing and exciting when a single InAs QD interacts with a single photon. Studying this interaction can not only help answer basic questions such as how a photonic qubit can be converted to a spin qubit for quantum computing^3,4,20^, but also yields novel device concepts such as single quantum dot single photon detectors (SQDSPDs)^21,22^. Recently, many efforts have been directed to the integration of InAs/GaAs QDs on silicon substrates and very promising results have been shown for photodetection and laser applications^23–26^. These efforts pave the way for interfacing InAs/GaAs QDs with even wider applications already developed and matured on the silicon platform.

Two-photon absorption (TPA) is a common optical nonlinear effect that occurs when semiconductors interact with high intensity light in the MW/cm^2^ to GW/cm^2^ range. This can result in dramatic changes in the optical responses of InAs/GaAs QDs as they relate to the above applications^27,28^. TPA is also widely used in various fields such as spectroscopy-based materials characterization^29,30^, biological fluorescent labeling and imaging^31,32^, solar energy harvesting by intermediate-band solar cells (IBSCs)^33,34^, and optical limiting for sensitive detector protection^35–38^. In general, there are three main mechanisms through which two photons can be absorbed by the semiconductor: direct two-photon absorption, indirect (two-step or sequential) two-photon absorption, and absorption via Auger processes. Direct two-photon absorption refers to the simultaneous absorption of two photons with their sum energy equal to or larger than the bandgap of the semiconductor, and it does not require the participation of real intermediate energy states for the transition while the other two mechanisms do.

Previously, TPA has been investigated in the InAs/GaAs QDs system for both device applications and fundamental physics studies. For example, two-step, two-photon absorption (TS-TPA) enables photocurrent generation in in QD based IBSCs and quantum dot-in-a well infrared photodetectors^6,7,33,34^. In addition, TPA in InAs QDs was found in studies of, for example, spectral hole burning^39^, Rabi oscillations^40^, and up-converted luminescence^41^. For these, TPA was essentially achieved either by a two-step process or by a simultaneous two exciton excitation to form a biexciton, both requiring the presence of real intermediate states (e.g., either intermediate bands or single exciton states) to fulfil the transition. In contrast, direct TPA is a more generic TPA process that occurs even in semiconductors without intermediate states. The magnitude of direct TPA is mainly related to the intrinsic density of states (DOSs) of the conduction and valence bands of the semiconductor rather than the less controllable DOSs of intermediate states, making direct TPA a more predictable process than the other TPA processes. In addition, direct TPA is a fast and coherent process while the other two are generally incoherent processes^42^. Therefore, direct TPA may provide a possible means for coherent manipulation of charges in semiconductors. Moreover, excitation by direct TPA allows optical access to “dark” states in QDs that are usually inaccessible to single-photon excitations^43^, and also allows photoluminescence (PL) studies using band-edge excitations which is challenging with single-photon excitation due to the difficulty in filtering out the relatively strong excitation light from the emitted light similar in energy.

In this paper, we present a systematic photoluminescence intensity study of MBE grown self-assembled InAs/GaAs QDs. Power dependent PL measurements were performed on ensembles of and single InAs/GaAs QDs by using one-photon (1 hν) and two-photon (2 hν) excitations. A quadratic power-law dependence of PL intensity was found on an ensemble of QDs with 2 hν excitation, which provided clear evidence for the occurrence of direct TPA in the QDs. To clarify the TPA channels, photoluminescence excitation (PLE) measurements were also conducted. A prominent peak corresponding to the ground state of the QDs was revealed in the PLE spectrum, which further confirmed the occurrence of direct TPA in the QDs. To the best of our knowledge, direct TPA in InAs QDs with excitation near its half band-gap has not been reported before. Thus, our work helps fill this gap and provides valuable insight into the direct TPA process that occurs on the versatile InAs/GaAs QDs platform for high intensity light excitations.

## Experimental

The self-assembled InAs/GaAs QDs were formed through Stranski–Krastanow (S-K) strain relaxation by MBE. Semi-insulating GaAs (100) substrates were used to grow on. Following a standard cleaning through heating above the native oxide desorption temperature, a 500 nm GaAs buffer layer was grown at 584 °C. Then 1.7 monolayers (MLs) of InAs were deposited at a rate of 0.022 ML/s at 460 °C. During the InAs deposition, the sample rotation was interrupted and the manipulator was tilted by 5° from its optimum position. As a result, a gradient in density and size of InAs QDs was formed due to the variation of the indium flux across the sample surface. The sample was then annealed for 40 s at 460 °C before a 150 nm GaAs cap layer was grown, on top of which another layer of InAs QDs was grown with the same deposition method for atomic force microscopy (AFM) study. The density and size distribution created by this gradient resulted in a wide range of luminescent energies of the QDs for study, including very high energy QDs above 1.24 eV. This energy range provides enhanced sensitivity of the silicon charge-coupled device (Si-CCD) detector such that very low signals can be studied spectroscopically. Additionally, the gradient resulted in regions of the sample where the QD density was low enough to identify single dots in the micro-PL system. This ultimately provides for a method to distinguish different absorption channels such as InAs QDs, InAs wetting layer (WL), and GaAs matrix, as discussed below.

**Figure 1:**
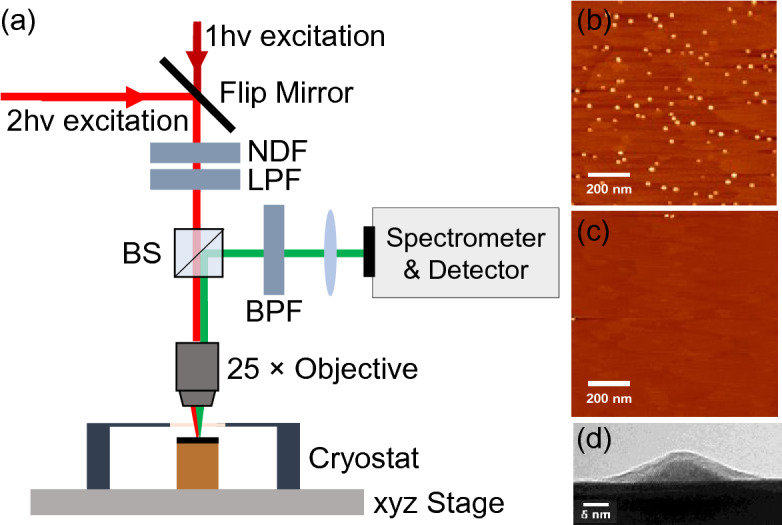
(**a**) Schematic diagram of the PL measurement system. NDF, LPF, BS, and BPF represent the neutral density filter, long-pass filter, beam splitter, and band-pass filter, respectively. (**b**,**c**) Representative AFM images of an area populated with InAs QDs (**b**) and an area almost clear of QDs (i.e. “WL only” area) (**c**). (**d**) Typical transmission electron microscopy (TEM) image showing the profile of an InAs SQD, where contrasts with different brightness from the bottom to the top represent the GaAs substrate, lens-shaped InAs QD, amorphization due to sample preparation, and amorphous carbon of the TEM grid, respectively.

Photoluminescence was collected using a Horiba LabRAM HR800 micro-PL system. A He–Ne laser emitting at 633 nm with a maximum power of I_10_ ~ 6 mW (excitation intensity ~ 12 kW/cm^2^) was used as the 1 hν excitation source. Here we use the first subscript “1” to denote 1 hν excitation, and it would become “2” for 2 hν excitation. The second subscript “0” indicates that the maximum power level of the laser was used for the measurement. A schematic diagram of the system is shown in Fig. 1a. A 0.75-m spectrometer and a thermoelectrically cooled Si-CCD are used to detect the PL signal. The samples were kept either at 77 K for the ensemble PL measurements or at 5 K for the SQD PL measurements in a continuous flow cryostat. For 2 hν excitation, linearly polarized 100 fs laser pulses (250 kHz repetition rate) generated from an optical parametric amplifier (Coherent OPA9850) connected with a regenerative amplifier (Coherent RegA9050) were guided into the LabRAM system. The output from the optical parametric amplifier (OPA) could be tuned from 0.517 to 0.775 eV. Long-pass filters were put in the excitation path to avoid any 2 hν excitation above the GaAs bandgap. The maximum average power of the femtosecond (fs) laser was ~ 5 mW (I_20_), corresponding to a pulse energy of 20 nJ and a peak power of 200 kW. The laser beam was focused onto the sample with a 25× reflective objective (0.4 N.A.) and the PL was collected by the same objective. The maximum 2 hν excitation intensity at the sample was about 400 GW/cm^2^.

## Results and discussion

Figure 1b,c are representative AFM images (1 × 1 µm^2^) of a QDs ensemble area and a “WL only” area, respectively. The QDs were found to have an areal density of ~ 1 × 10^10^ cm^−2^ with an average height and diameter of 2.67 ± 0.61 nm and 23.07 ± 3.18 nm, respectively. In the “WL only” area, the InAs deposition was below the critical thickness and thus no QDs were formed. In Fig. 1c, three SQDs are visible on the boundaries of the “WL only” area. Owing to the gradient of the QD density, such SQDs could easily be identified and optically isolated for SQD PL study. Figure 1d shows a typical side-view TEM image of a lens-shaped InAs QD found in this study. This breaking of the geometric symmetry along the growth direction helps relax the optical selection rules, making the QDs more sensitive to normal incident and in-plane polarized lights than quantum wells (QWs) for intraband transitions^44,45^.

1 hν excitation power-dependent PL study was first performed on an ensemble of InAs QDs and a SQD to understand the fundamental optical properties of the QDs and the photocarrier dynamics of the QD/WL/GaAs system. For this, PL was recorded while the 1 hν excitation power was varied by two to four orders of magnitude. With low-power 1 hν excitations, carriers were expected to be generated mainly in the GaAs matrix due to its large absorption volume, and then quickly relax into the confined QD ground states and recombine there radiatively (emitting photons) or non-radiatively (without photon emission). With increasing excitation power, carriers first fill up the ground states and then subsequently fill higher energy states (excited states) of the QDs. For sufficiently strong excitations, population of the WL and finally the GaAs band-edge states might be achieved. This state filling would result in the observation of PL initially from the QDs then from the WL and eventually from the GaAs band-edge with increasing excitation power.

**Figure 2:**
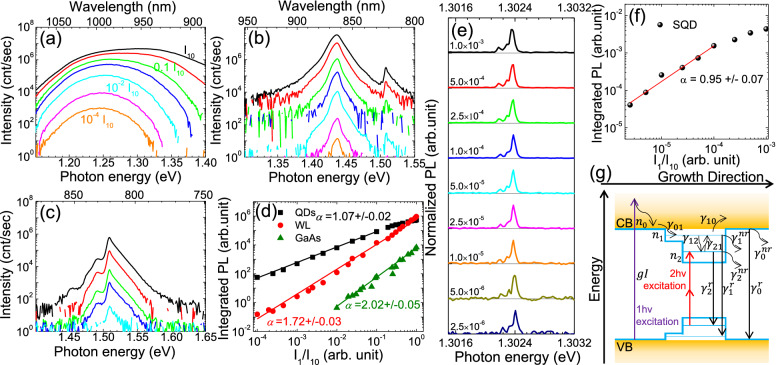
1 hν excitation power-dependent PL spectra. PL spectra at 77 K: (**a**) QD ensemble; (**b**) “WL only” sample; (**c**) GaAs wafer. The excitation power was varied from 10^−4^I_10_ to I_10_ as labeled in (**a**). (**d**) Integrated PL intensity vs normalized 1 hν excitation power for the data in (**a**)–(**c**). The straight lines are best fits to the data with a function of I_PL_ = bI^α^, where b and α are fitting parameters. PL spectra of single QDs at 5 K: (**e**) Power-dependent SQD spectra showing the ground state excitonic emissions. The numbers indicate the power ratio between the excitation power and the maximum laser power which equals to an I_10_ ~ 6 mW. The PL spectra were normalized to the corresponding peak intensities at 1.3024 eV. (**f**) Integrated PL intensity vs. excitation power ratio. (**g**) Schematic diagram for the three-level rate equation model. Straight arrows denote optical transitions while curve arrows denote relaxation or capture processes.

Figure 2a shows typical PL spectra obtained from an ensemble of QDs. The excitation power was varied from 10^−4^I_10_ to I_10_, as marked by different colors. The QD PL peaked at 1.24 eV with a full width half maximum (FWHM) of 62.4 meV and at an excitation power of 0.6 µW (orange curve). As the power increased gradually to the maximum power, the QD emission (black curve) blue shifted monotonically to 1.30 eV and broadened to a FWHM of 122 meV. This blue shift is a characteristic of the state filling in the QDs, as discussed above. However, the excited states emission was not well resolved here due to the very broad size distribution of the QDs and for this reason we limited the measurements of the PL of the QDs ensemble to 77 K. To fully understand the photocarrier dynamics of the QD/WL/GaAs system, we also measured the PL from a “WL only” sample (Fig. 2b), and from a semi-insulating GaAs wafer (Fig. 2c). For the “WL only” sample (Fig. 1b), when the excitation power was greater than 0.1I_10_, the GaAs band-edge emission at 1.51 eV became significant, which could again be ascribed to the state filling effect. In Fig. 2d, we plot the PL intensities integrated from 1.20 to 1.30 eV, 1.42–1.45 eV, and 1.47–1.53 eV for the QDs ensemble (black squares), the “WL only” sample (red circles), and the GaAs wafer (green triangles), respectively, as a function of excitation power fraction (I_1_/I_10_). All the data could be well described by a power law of the form I_PL_ ~ I^α^ with an α equal to 1.07, 1.72 and 2.02 for the QDs, WL, and GaAs band-edge emission, respectively. Below, we adopt a simple three-level rate equation model to rationalize this power law behavior.

Figure 2g shows a schematic diagram of the energy levels of the QDs/WL/GaAs system and the processes that we take into account in the model. For the WL and GaAs samples we can build similar models and reach essentially the same conclusions. This model tracks the time evolution of the free carrier (e.g., electron) densities in the GaAs matrix (${n}_{0}$), the WL (${n}_{1}$) states and the QD (${n}_{2}$) states. This does not necessarily exclude excitons, but for simplicity, we ignore any excitonic effects in the WL and GaAs levels and focus on the dynamics of electrons. The processes, each represented by a transition rate, are: carrier capture by the WL from the GaAs matrix (${\gamma }_{01}$ ), capture by the QDs from the WL (${\gamma }_{12}$ ); thermal escape from the WL to the matrix (${\gamma }_{10}$ ), thermal escape from the QDs to the WL (${\gamma }_{21}$ ); and finally radiative (${\gamma }_{0}^{r}, {\gamma }_{1}^{r}, {\gamma }_{2}^{r}$ ) and non-radiative decay (${\gamma }_{0}^{nr}, {\gamma }_{1}^{nr}, {\gamma }_{2}^{nr}$ ) from each level. With 633 nm excitation, carriers are generated in the matrix with a rate $gI$, where $I$ is the excitation power and $g$ is the generation coefficient. At low excitation powers, we assume the state filling effect has not taken place, and the rate equations for ${n}_{0}, {n}_{1}, {n}_{2}$ can be written as:

1 $$\text{GaAs}{:}\quad \frac{d{n}_{0}}{dt}=gI+{n}_{1}{\gamma }_{10}- {n}_{0}{\gamma }_{01}-{n}_{0}{\gamma }_{0}^{r}- {n}_{0}{\gamma }_{0}^{nr}$$

2 $$\text{WL}{:}\quad\frac{d{n}_{1}}{dt}={n}_{0}{\gamma }_{01}+{n}_{2}{\gamma }_{21}- {n}_{1}{\gamma }_{10}- {n}_{1}{\gamma }_{12}-{n}_{1}{\gamma }_{1}^{r}- {n}_{1}{\gamma }_{1}^{nr}$$

3 $$\text{QDs}{:}\quad  \frac{d{n}_{2}}{dt} = {n}_{1}{\gamma }_{12}-{n}_{2}{\gamma }_{21}-{n}_{2}{\gamma }_{2}^{r}-{n}_{2}{\gamma }_{2}^{nr}$$

Based on our experimental conditions, two assumptions can be made. Firstly, at our experimental temperature of 77 K, the thermal energy ${k}_{B}T$ (6.6 meV) is still an order of magnitude less than the energy level spacing in the conduction band (30–70 meV)^46^ or the conduction band offset between the WL layer and the GaAs substrate, where ${k}_{B}$ is the Boltzmann constant and $T$ is the temperature. Therefore, the thermal escape terms, ${n}_{1}{\gamma }_{10}$ and ${n}_{2}{\gamma }_{21}$ , are negligible. Secondly, at low excitation powers, no WL or GaAs emission is observed whereas the QD emission is strong. Therefore, we assume that the probability of carriers being captured by the QDs is much higher than that of carriers that radiatively recombine in WL or GaAs, namely ${n}_{0}{\gamma }_{01}\gg {n}_{0}{\gamma }_{0}^{r}$ , ${n}_{1}{\gamma }_{12}\gg {n}_{1}{\gamma }_{1}^{r}$.With these two assumptions and under steady state condition, we can derive from the above equations:

4 $$gI\approx {n}_{0}{\gamma }_{01}+{n}_{0}{\gamma }_{0}^{nr} \Rightarrow  {n}_{0}\approx \frac{gI}{{\gamma }_{01}+{\gamma }_{0}^{nr}} \sim I$$

5 $${n}_{0}{\gamma }_{01}\approx {n}_{1}{\gamma }_{12}+{n}_{1}{\gamma }_{1}^{nr}  \Rightarrow {n}_{1}\approx {n}_{0}\frac{{\gamma }_{01}}{{\gamma }_{12}+{\gamma }_{1}^{nr}} \sim I$$

6 $${n}_{1}{\gamma }_{12}\approx {n}_{2}{\gamma }_{2}^{r}+{n}_{2}{\gamma }_{2}^{nr}$$

In Eq. (6), if radiative recombination dominates, then we have

7 $${\text{n}}_{2}{\upgamma }_{2}^{\text{r}}\approx {\text{n}}_{1}{\upgamma }_{12} \Rightarrow {\text{n}}_{2}\approx {\text{n}}_{1}\frac{{\upgamma }_{12}}{{\upgamma }_{2}^{\text{r}}}\sim \text{I}$$

8 $${\text{n}}_{2}{\upgamma }_{2}^{\text{n}\text{r}}\approx {\text{n}}_{1}{\upgamma }_{12} \Rightarrow {\text{n}}_{2}\approx {\text{n}}_{1}\frac{{\upgamma }_{12}}{{\upgamma }_{2}^{\text{n}\text{r}}}\sim \text{I}$$

If non-radiative recombination dominates, then.

Equations 4, 5, 7, and 8 indicate that the electron density for each level is proportional to the excitation power. The relationship between the hole densities ${p}_{0}, {p}_{1},{ p}_{2}$ and the excitation power can be derived similarly, and they also have a linear dependence on the excitation power. Below we will derive the relationship between the PL intensity and excitation power. The PL intensity of each level $i (i=0, 1, 2)$ can be written as:

9 $$P{L}_{i} \sim {F}_{i}{n}_{i}{p}_{i}+{C}_{i}{n}_{i}\sim {F}_{i}{n}_{i}^{2}+{C}_{i}{n}_{i}$$

Here we treat ${p}_{i}\sim {n}_{i}$ since they have the same $I$ dependence. ${F}_{i}{n}_{i}{p}_{i}$ represents the free carrier (or uncorrelated electron and hole) recombination with a coefficient ${F}_{i}$, while ${C}_{i}{n}_{i}$ is the correlated electron–hole recombination term with a coefficient ${C}_{i}$^47,48^. If free carrier recombination dominates, $P{L}_{i}\sim {F}_{i}{n}_{i}^{2}\sim {I}^{2}$; if correlated electron–hole recombination dominates, $P{L}_{i}\sim {C}_{i}{n}_{i}\sim I$. In QDs, carriers are strongly confined, and correlated electron–hole recombination dominates. Therefore, $P{L}_{QDs}\sim I$. In the GaAs matrix, however, the photogenerated electrons and holes, due to lack of spatial confinement, may diffuse in random directions and lose the correlation, and consequently recombine as free carriers with a luminescent intensity, $P{L}_{GaAs}\sim {I}^{2}$. The WL provides a quasi-one-dimensional confinement, where carriers neither are spatially confined as much as they are in the QDs nor can move as freely as in the matrix. We therefore assume that PL intensity scales as $P{L}_{WL}\sim {I}^{\alpha }$ with $1<\alpha <2$ .

The linear power dependence of the QD ensemble in Fig. 2d implies correlated, excitonic recombination within the QDs. To further confirm this excitonic recombination behavior, we performed power-dependent PL studies on a SQD at 5 K. The elimination of broad size distribution and the reduction in measurement temperature allowed us to clearly resolve the emission peaks of SQD in the PL spectra. Prior to power-dependent measurements, we followed a procedure established by us to ensure that only a SQD was excited and studied. (see Supplementary Information (SI) for details about the procedure). The original PL spectra are reported in Fig. S1 in SI. In Fig. 2e, the normalized SQD PL spectra at different excitation power ratios are plotted as a function of photon energy. A main peak at 1.3024 eV is clearly identified and the emission takes on a similar spectral shape at all excitation power ratios, suggesting that this emission was indeed from a SQD. The power-dependent integrated intensity of the GS PL peak was obtained from Fig. S1 and is presented in Fig. 2f. The straight line is a power law fit to the intensities. Data points above 10^–4^ I_1_/I_10_ were excluded from the fitting due to their apparent deviation from the linear region caused probably by the saturation effect. The linear dependence shown in Fig. 2f helped rule out the assignment of GS PL peaks to biexcitonic recombination, and confirmed that the linear PL power dependence indeed resulted from the excitonic recombination in the QD.

**Figure 3:**
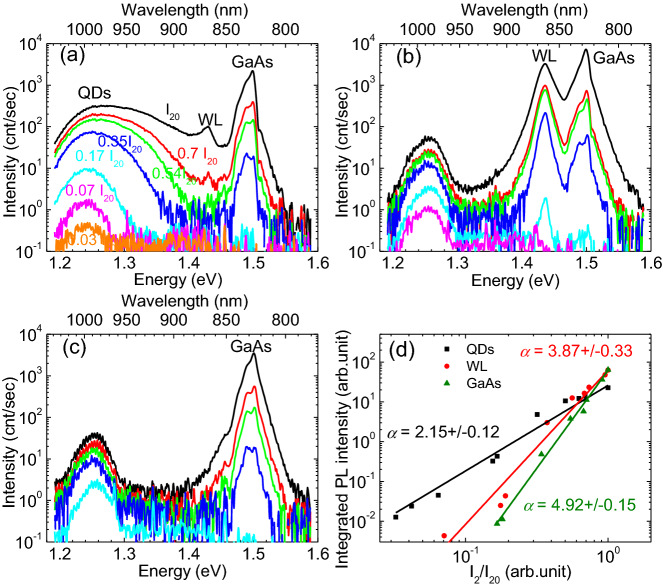
Power-dependent PL spectra with a 2 hν excitation energy of 1.24 eV: (**a**) QD ensemble; (**b**) “WL only” sample; (**c**) GaAs wafer. The excitation power varied from 0.03I_20_ to I_20_ as labeled in (**a**). (**d**) Integrated PL intensities extracted from (**a**)–(**c**) as a function of normalized 2 hν excitation power for the QDs ensemble. The straight lines are power-law fits to the data.

After establishing the fundamental optical properties of InAs QDs by 1 hν excited PL, we now turn to the 2 hν excited PL studies, where three excitation energies were chosen, namely 1.240 eV, 1.432 eV, 1.538 eV, corresponding to two-photon excitation into the QDs ensemble ground state, the WL state, and continuum states above the GaAs band-edge, respectively. Figure 3a–c show the PL spectra of QDs ensemble, “WL only”, and GaAs samples, respectively, recorded with a 2 hν excitation energy of 1.240 eV. For the other two excitation energies, the PL shape and trend with excitation power are similar to what are shown in Fig. 3. It should be noted that while the emission around 1.250 eV in Fig. 3a was mainly from the QDs, any intensity in this spectral region in Fig. 3b,c should not be associated with QD emission, since these samples did not contain any QDs, which was confirmed by the 1 hν excited PL. We noticed that the low-energy peak in Fig. 3b,c always appeared at twice of the excitation photon energy and moved with the excitation energy. Thus, we asserted that this signal stemmed either from second harmonic generation (SHG) or from two-photon induced luminescence (TPL) which is the light emission resulting from the recombination of two-photon excited electrons in subbandgap states with holes in the valence band. Given the small density of subbandgap states in the GaAs buffer layer, we could exclude the possibility of TPL and associate it with the SHG near the GaAs/vacuum interface, as suggested by previous studies^49^. We also note that the WL and GaAs band-edge PL peaks shown in Fig. 3b,c could not originate from TPA, since the 2 hν energy (1.24 eV) used here was smaller than the bandgap energies of both the WL and the GaAs substrate. We hypothesize that these PL peaks might result from the absorption of the third-harmonic generation (THG) light generated near the GaAs/vacuum interface by the WL and GaAs substrate^50–53^. We will discuss this further later. Similar peaks also appeared in Fig. 3a, which presumably have the same origin as those in Fig. 3b,c. It is noticeable that the intensity of WL peaks in Fig. 3a was at least one order of magnitude lower than that of the WL peaks shown in Fig. 3b at the same excitation power. This reduced intensity is likely due to the carrier transfer from the WL states to the QD states. The interplay between the WL states and the QD states may be eliminated by overgrowing a monolayer of AlAs on the QDs, as suggested by a recent report^54^.

To deduce the net 2 hν excited PL from the QDs, we took the SHG from the GaAs wafer (Fig. 3c) as a baseline and subtracted it from the PL spectra of the QDs ensemble (Fig. 3a). The same procedure was carried out when the SHG peak overlapped the WL PL with a 2 hν excitation energy of 1.432 eV. Figure 3d presents the power-law fits to the integrated intensities of the QDs ground state PL (1.20–1.30 eV), the WL PL (1.42–1.45 eV), and the GaAs PL (1.47–1.53 eV), with a 2 hν excitation energy of 1.24 eV. The power-law fitting yielded an exponent, α, of 2.15, 3.87, and 4.92 for these samples, respectively. Table 1 summarizes the extracted exponents for all three 2 hν excitation energies that we used for each sample. The exponents corresponding to the WL and GaAs peaks of the QDs ensemble sample (Fig. 3a) can be found in Table S1 in SI.

**Table 1:** Extracted power law exponents for three 2 hν excitation energies.

Sample	1.240 eV	1.432 eV	1.538 eV
QDs ensemble	2.15 ± 0.12	2.16 ± 0.14	1.89 ± 0.15
WL only	3.87 ± 0.28	3.20 ± 0.29	2.91 ± 0.10
GaAs wafer	4.92 ± 0.15	5.96 ± 0.26	5.81 ± 0.55

It can be concluded from Table 1 that the integrated PL intensities of the QDs ensemble exhibited a quadratic power dependence for all three 2 hν excitation energies. This quadratic dependence was also reported on other QDs such as CdSe and CdS QDs, and was generally considered as direct evidence of 2 hν absorption for semiconductor QDs^55,56^. On the other hand, a power exponent of less than two was usually ascribed to TS-TPA occurring in InAs/GaAs self-assembled QDs^41,57^. Thus, we concluded that the QDs PL shown in Fig. 3a most likely resulted from direct 2 hν excitation.

**Figure 4:**
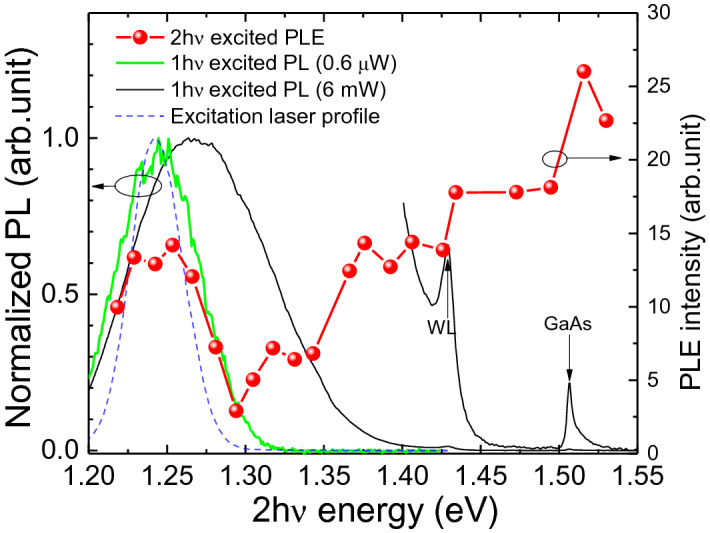
TP-PLE spectrum together with the low-power (green curve) and high-power (black curve) 1 hν excited PL spectra. The high-power spectrum beyond 1.4 eV is multiplied by a factor of 50 and replotted to show the band-edge emission from WL and GaAs. The intensity squared of the frequency-doubled excitation laser spectrum (blue dashed curve) with an excitation energy of 0.62 eV (equivalent 2 hν energy of 1.24 eV) is also plotted to illustrate the spectral width of the excitation laser.

The direct 2 hν absorption in the QDs is further supported by the resonant peak appearing in the two-photon photoluminescence excitation (TP-PLE) spectrum shown in Fig. 4. PLE spectroscopy is a useful tool to reveal the optical absorption channels of semiconductors. The TP-PLE measurement was performed by scanning the 2 hν excitation energy from 1.22 eV to 1.53 eV with a constant power while fixing the collection energy at ~ 1.24 eV. The integrated QD PL (1.20–1.30 eV) from the ensemble is plotted as a function of the 2 hν excitation energy (red dots) together with the low-power (green curve) and high-power (black curve) 1 hν excited PL spectra in Fig. 4. A clear resonant peak was observed in the PLE spectrum when the 2 hν excitation energy coincided with the QDs ground state (1.24 eV), indicative of resonant direct 2 hν excitation in the QDs. We note that the resonant peak intensity might have ~ 10% contribution from the SHG signal as discussed above (Fig. 3), and removing this small contribution would not change the resonant nature of the peak shown in Fig. 4. Notably, this resonant peak correlates well with the low-power 1 hν excited PL peak, which implies that the 2 hν and 1 hν excitations shared a common radiative recombination channel at low excitation levels.

Away from the resonant peak, the TP-PLE signal reaches a minimum at 1.29 eV, before increasing almost monotonically with the 2 hν excitation energy, and surpassing the resonant peak intensity at a 2 hν energy of ~ 1.43 eV, the bandgap energy of WL. The minimum at 1.29 eV suggests that, for above-bandgap two-photon excitations, detrimental effects caused by TPA in device applications may be mitigated by offsetting the laser wavelength slightly to the ground-state resonant peak of the QD ensemble. This can be realized either by tuning the working laser wavelength or by changing the QD size through growth control. The monotonical increase of the QDs PLE intensity can be rationalized by the gradual increase of the 2 hν absorption of the WL and GaAs substrate as the 2 hν excitation energy approached their respective band-edge energies. Step-like features are clearly seen around these energies, indicating the onset of the above-bandgap TPA in WL or GaAs. We remark that the TP-PLE intensity due to the two-photon absorption of GaAs is only ~ 2 times of the resonant peak intensity, which is incommensurate to the large difference in DOSs between the bulk GaAs and the QDs. We suggest that this may be related to the enhanced TPA in QDs due to strong quantum confinement^58^. Similar to the scenario in 1 hν excitation PL, carriers generated by 2 hν absorption of the WL and GaAs substrate would relax to the lower-lying energy states of QDs, and add to the radiative recombination of the carriers generated by 2 hν absorption in the QDs, giving rise to enhanced PL intensity.

**Figure 5:**
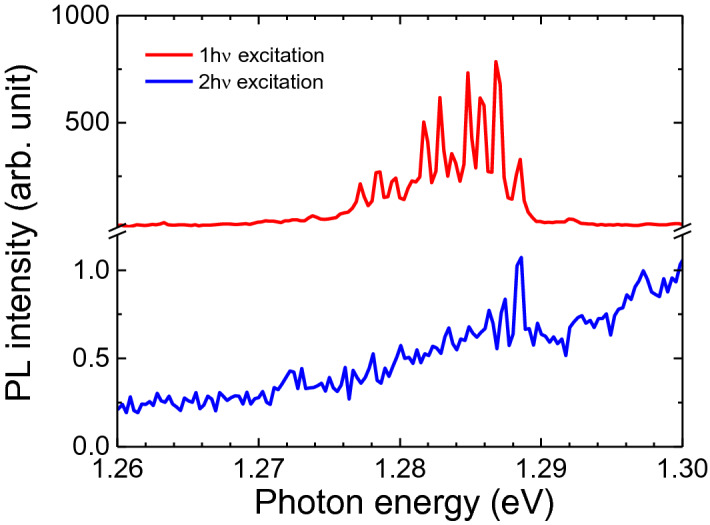
Comparison of the GS SQD PL excited by 1 hν (red) and 2 hν (blue) sources at 5 K.

To further scrutinize the resonant peak correlation of the QDs, we adopted a similar strategy as in Fig. 2e by performing the 1 hν and 2 hν excited PL measurements on a SQD at 5 K. Figure 5 demonstrates a correlation of single quantum dot emission peaks, well identified by their extremely narrow linewidth, under both 1 hν and 2 hν excitation. These are found by moving to a low dot density region, on the order of 1 dot per 4 µm^2^, on the gradient sample, like in Fig. 1c. At 5 K, SQD emission shows a clear alignment of the higher energy lines between both excitation conditions, confirming the existence of a shared recombination channel. We note that the 2 hν excitation was performed using a non-resonant 2 hν energy of ~ 1.35 eV to avoid interacting with the SHG signal. Recently, similar peak correlation was also found in colloidal CdSe/ZnS QDs^59^.

We now turn to providing an explanation for the large exponents (3–6) reported in Table 1 for the WL and GaAs samples in 2 hν excitation measurements. It has been well established in the literature that SHG and THG may be enhanced, compared to those in the bulk, near the dielectric interface due to breaking of either the lattice symmetry or the focal volume symmetry (the integrity of the laser interaction volume with a length of confocal parameter), or both^49–52^, and this effect has been extensively explored for bio- and medical imaging^60–62^. In our experiments, the light intensity was above 100 GW/cm^2^. Thus, efficient SHG and THG are likely to occur near the GaAs/vacuum interface. Indeed, we have observed SHG signals in PL results exhibited in Fig. 3. As we extended the PL collection to the THG energy range, THG signals with comparable or even higher magnitude than that of SHG signals were observed when exciting the GaAs wafer at an energy less than half of its bandgap (see Fig. S2 in the SI). Based on this observation, we then hypothesize that an exponent of ~ 6 for GaAs (Table 1) may result from a two-step process, in which THG photons are first generated near the GaAs interface and then absorbed by the WL and GaAs bulk to bring about PL. In the first step, the generated THG intensity ${I}_{THG}$ is proportional to ${I}_{2}^{3}$, where ${I}_{2}$ is the 2 hν excitation power. By recalling that the PL intensity of GaAs by 1 hν excitation follows a quadratic dependence on the power (Fig. 2d), one then reaches a PL intensity dependence of the form of ${cI}_{2}^{6}$, with $c$ being a proportionality factor. A smaller exponent (< 4) for the WL can again be accounted for by considering a mixture of excitonic and free-carrier recombinations in the WL due to its two-dimensional nature. We note that the carriers generated by the THG photons in the WL and GaAs can, in principle, also contribute to the 2 hν excited QDs PL (e.g. in Fig. 3a) through relaxation into the QD energy states, as in the 1 hν excitation case. However, we do not consider this contribution to be significant for following reasons. First, when measuring the 2 hν excited QDs PL (e.g. in Fig. 3) we moved the focal point to a position slightly below the sample surface to maximize the PL signal. This resulted in a weakening of the interface enhancement of the THG. In addition, the QDs PL dependence on excitation intensity showed a clear quadratic behavior (Fig. 3) whereas a significant contribution from the carriers generated by the THG absorption would have led to a cubic dependence of the QDs PL intensity on excitation power. Though the simple model described above provides an explanation for the large exponents associated with the WL and GaAs samples, we are aware that other high optical field related effects may also give rise to such high-order optical nonlinearity. For example, Keldysh theory suggests that these high order nonlinearities may be taken as a signature of the breakdown of the regime of traditional nonlinear optics and could be interpreted as manifestations of strong-field multiphoton ionization (MPI) in semicondcutors^63^. The Keldysh model distinguishes between two different regimes of MPI. For Keldysh parameters exceeding unity, multiple photons are absorbed from the laser beam to excite an electron from the valence to higher energy states in the conduction band. In this regime, the ionization rate depends on the laser intensity in a highly nonlinear manner. For Keldysh parameters below unity, tunneling ionization dominates, yielding a much weaker dependence of the photoionization rate on the laser intensity. In our case, we estimated a Keldysh parameter of 1.7, suggesting a regime of multiphoton ionization. A more detailed discussion can be found in the SI. Nevertheless, further experiments are needed to clarify the origin of these large exponents.

## Conclusion

In summary, we performed power-dependent PL studies on MBE-grown InAs/GaAs QDs ensembles. A quadratic power dependence of the 2 hν excited PL of the QDs ensemble together with the resonant emission shown on the TP-PLE spectrum ambiguously confirmed the occurrence of the direct TPA in InAs/GaAs QDs. The TP-PLE spectrum also revealed a minimum of TPA for two-photon energies larger than the QD bandgap, which suggests detrimental TPA in device applications may be mitigated by tuning the working laser wavelength slightly away from the resonant two-photon absorption peak of the QDs. We also proposed a three-level rate equation model to rationalize the carrier dynamics in the QDs/WL/GaAs system and the results of 1 hν excited power-dependent PL. Furthermore, we observed higher order optical nonlinear effects on “WL only” and GaAs samples, which was explained by a model based on third-harmonic generation and absorption. Our results provide valuable insight for understanding both linear and nonlinear optical processes in the InAs/GaAs QD system, a versatile and important platform for many fundamental and device application research frontiers such as quantum information, quantum computing, and integrated photonics.

## Supplementary information

Supplementary file1 (DOCX 132 kb)
