# Supplementary material for: Photoluminescence of InAs/GaAs quantum dots under direct two-photon excitation
Source: Sci Rep. 2020 Jul 2;10:10930. doi: 10.1038/s41598-020-67961-z (PMC7331710; doi:10.1038/s41598-020-67961-z)
Supplement: Supplementary file 1 — Supplementary file1 (DOCX 132 kb) [file 41598_2020_67961_MOESM1_ESM.docx]

Supplementary Information

**Photoluminescence of InAs/GaAs quantum dots under direct two-photon excitation**

Xian Hu^1^, Yang Zhang^1*^, Dorel Guzun^1^, Morgan E. Ware^2^, Yuriy I. Mazur^1*^, Christoph Lienau^3^, Gregory J. Salamo^1^

^1^ Institute for Nanoscience and Engineering, University of Arkansas, Fayetteville, AR, 72701, USA

^2^Department of Electrical Engineering, University of Arkansas, Fayetteville, AR, 72701, USA

^3^Institute of Physics and Center of Interface Science, Carl von Ossietzky University, Oldenburg 26129, Germany

Corresponding authors: yangzh08@gmail.com; [ymazur@uark.edu](mailto:ymazur@uark.edu)

Procedure to determine the presence of a single quantum dot (SQD)

For the preliminary location of a SQD, the 1hν excitation laser (633 nm) was attenuated to a very low power (~1 nW) and scanned by moving the xyz stage of the Horiba LabTAM HR800 system (Fig. 1a) through an area of low QD density, like the one shown in Fig. 1c in the main text. Once a single emission peak at the ground state (~1.24 eV) is caught by the CCD camera, a more careful scan is performed in an area (2 μm × 2 μm) centered at the highest intensity location of this single peak. The edge length of this area is slightly larger than the laser beam diameter (1.93 μm). Therefore, if this is the sole emission peak showing up in this area, we conclude that there is only one QD in this area. Then we continue with the power-dependent PL measurements, as presented in Fig. 2e.

Power-dependent PL spectra on a SQD at 5 K


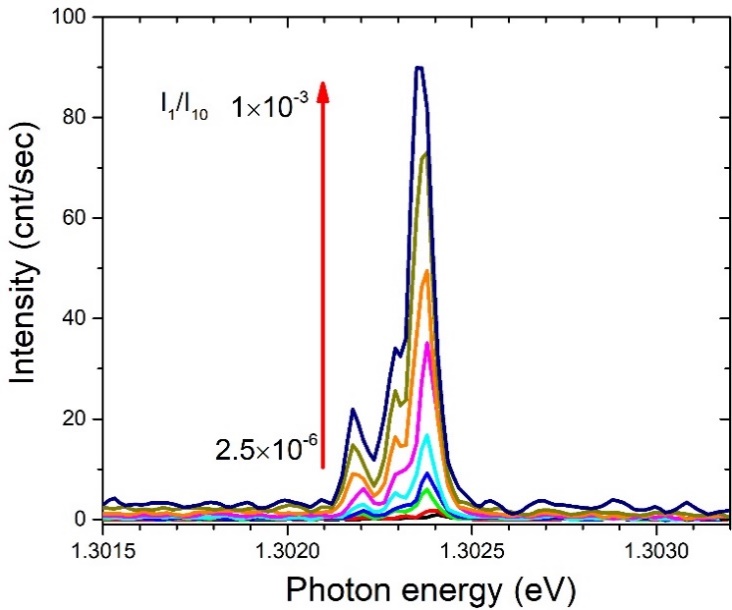
After pinpointing a SQD using the procedure detailed above, we performed PL measurement on this SQD by using 1hν excitation power ratios (I_1_/I_10_) of 2.5×10^-6^, 5×10^-6^, 1×10^-5^, 2.5×10^-5^, 5×10^-5^, 1×10^-4^, 2.5×10^-4^, 5×10^-4^, and 1×10^-3^. The absolute PL intensity shows a continuous increase with the excitation power, indicating that the ground state of the SQD was not saturated even at an excitation power of 6 µW. The intensity of the main peak at 1.3024 eV was integrated and plotted as a function of power ratio in Fig. 2f.

Figure S1. Power-dependent PL spectra recorded on a SQD at 5 K. The numbers along with the red arrow indicate the change of the 1hν excitation power ratio. I1 denotes the absolute power and I_10_ denotes the maximum laser power (6 mW).

Power-law exponents extracted from QDs ensemble, “WL only”, and GaAs wafer samples

In addition to the exponents reported in Tab. 1 in the main text, here we include also the “WL” and “GaAs” exponents extracted from the QDs ensemble sample as two rows at the bottom in Tab. S1. At three 2hν excitation energies, the exponents corresponding to the WL and GaAs peaks of the QDs ensemble sample, in general, agree with those extracted from the “WL only” and GaAs wafer samples. The slight increase of the exponents in the “WL (QDs ensemble)” row and the slight decrease of the exponents in the “GaAs (QDs ensemble)” row, with respect to the exponents at each excitation energy for “WL only” and GaAs wafer samples, might be attributed to the carrier transfer between the QDs, WL, and GaAs in the QDs ensemble sample, which could be clarified in future studies.

| Sample | 1.240 eV | 1.432 eV | 1.538 eV |
| --- | --- | --- | --- |
| QDs ensemble | 2.15±0.12 | 2.16±0.14 | 1.89±0.15 |
| WL only | 3.87±0.28 | 3.20±0.29 | 2.91±0.10 |
| GaAs wafer | 4.92±0.15 | 5.96±0.26 | 5.81±0.55 |
| WL (QDs ensemble) | 4.05±0.77 | 3.70±0.54 | 4.13±0.86 |
| GaAs (QDs ensemble) | 3.75±0.19 | 5.88±0.38 | 5.46±0.68 |

Table S1. Power-law exponents extracted from the integrated PL data plotted as a function of excitation power ratio.

SHG and THG in the GaAs wafer

To investigate the possible origin of the ~6 exponent, we performed power-dependent PL measurements at 77 K on a GaAs wafer with a fs laser photon energy of ~0.64 eV. As we extended the spectral range of the PL collection to ~2.06 eV, a third peak (~1.9 eV) appeared on the spectra in addition to the SHG and GaAs band-edge peaks. Since the third peak energy is ~3 times of the excitation photon energy, it is reasonable to believe that this peak originated from the THG. The intensity of the THG peak appeared initially lower than that of the SHG peak at an excitation power of 0.05 μW. However, it increased rapidly with power and surpassed the intensity of the SHG peak at a power of ~5 mW, suggesting efficient THG in the GaAs wafer. Meanwhile, the emission peak from the GaAs band edge also increased with power, alongside the increase of the THG peak, which led us to hypothesize that the GaAs peak was a result of the absorption of the THG light.

Figure S2. Power dependent second-harmonic generation (SHG) and third-harmonic generation (THG) in GaAs wafer at 77 K. The excitation laser energy was ~0.64 eV. The peaks around 1.5 eV were the PL from GaAs wafer.

Large exponents in Fig. 3d explained by Keldysh effect

Recently, high orders of the optical nonlinearity have been reported, for instance, for mid-gap excitation of large band gap zinc oxide nanostructures with few-cycle laser pulses^1,2^.

These high order nonlinearities have been taken as a signature of the breakdown of the regime of traditional nonlinear optics. and were interpreted as manifestations of strong-field multiphoton ionization (MPI) in semiconductors, as predicted by Keldysh theory^3^. Essentially, the electric field of the excitation laser is strong enough to induce a periodic modulation of the bandgap^4^, causing electron tunneling from the conduction to the valence band and - thus - a light-driven current in the solid^5^.

The Keldysh model distinguishes between two different regimes of MPI. For Keldysh parameters exceeding unity, multiple photons are absorbed from the laser beam to excite an electron from the valence band to higher energy states in the conduction band. In this regime, the ionization rate depends on the laser intensity in a highly nonlinear manner. For Keldysh parameters below unity, tunneling ionization dominates, yielding a much weaker dependence of the photoionization rate on the laser intensity^6,7^. An improved version of the Keldysh model has indeed been used to account for high-order nonlinearities in midbandgap-excited MPL from ZnO nanostructures^1,8^. In gallium arside, the Keldysh model could explain polarization-dependent modulations in laser transmission arising from variations in the reduced mass of the charge carrier seen when exciting a semi-insulating GaAs(100) layer at 1900 nm laser wavelength with intensities in the 100 GW/cm^2^ regime. In our experiments, similar laser intensities are reached. In semiconductors, the Keldysh parameter may be estimated as

$\gamma=\frac{\omega_{L}}{eE_{0}}\sqrt{m^{*}E_{g}}$ (1)

where ω_L_, and E_0_ denote the angular frequency and field amplitude of the driving laser. For our 100-fs pulses, the peak intensity of 400 GW/cm^2^ corresponds to a field amplitude of 0.9 V/nm. Using an effective electron mass of m* = 0.07m_e_ and a bandgap energy of E_g_ = 1.5 eV, we estimate a Keldysh parameter of 1.7, well above the onset of strong-field tunneling but in a regime of strong MPI. This estimate suggests that the high-order optical nonlinearities observed for mid-bandgap excitation of PL from the wetting and GaAs buffer layer may be related to strong-field photoionization processes, likely to be affected by the complex dephasing and relaxation processes in these hybrid nanostructures.

References

1 Hyyti, J. *et al.* Field enhancement of multiphoton induced luminescence processes in ZnO nanorods. *J Phys D Appl Phys* **51**, 105306, https://doi.org/10.1088/1361-6463/aaaabe (2018).

2 Schmidt, S. *et al.* Distinguishing between ultrafast optical harmonic generation and multi-photon-induced luminescence from ZnO thin films by frequency-resolved interferometric autocorrelation microscopy. *Opt Express* **18**, 25016-25028, https://doi.org/10.1364/Oe.18.025016 (2010).

3 Keldysh, L. V. Ionization in the Field of a Strong Electromagnetic Wave. *Sov. Phys. JETP* **20**, 1307 (1965).

4 Kruchinin, S. Y., Krausz, F. & Yakovlev, V. S. Colloquium: Strong-field phenomena in periodic systems. *Rev Mod Phys* **90**, 021002, https://doi.org/10.1103/RevModPhys.90.021002 (2018).

5 Schiffrin, A. *et al.* Optical-field-induced current in dielectrics. *Nature* **493**, 70-74, https://doi.org/10.1038/nature11567 (2013).

6 Bormann, R., Gulde, M., Weismann, A., Yalunin, S. V. & Ropers, C. Tip-Enhanced Strong-Field Photoemission. *Phys Rev Lett* **105**, 147601, https://doi.org/10.1103/PhysRevLett.105.147601 (2010).

7 Piglosiewicz, B. *et al.* Carrier-envelope phase effects on the strong-field photoemission of electrons from metallic nanostructures. *Nat Photonics* **8**, 38-43, https://doi.org/10.1038/Nphoton.2013.288 (2014).

8 Mero, M., Liu, J., Rudolph, W., Ristau, D. & Starke, K. Scaling laws of femtosecond laser pulse induced breakdown in oxide films. *Phys Rev B* **71**, 115109, https://doi.org/10.1103/PhysRevB.71.115109 (2005).
